# RagC GTPase regulates mTOR to promote chemoresistance in senescence-like HepG2 cells

**DOI:** 10.3389/fphys.2022.949737

**Published:** 2022-10-04

**Authors:** Wei Jiang, Zhenglin Ou, Qin Zhu, Hongyan Zai

**Affiliations:** ^1^ Department of General Surgery, Xiangya Hospital, Central South University, Changsha, Hunan, China; ^2^ National Clinical Research Center for Geriatric Disorders, Central South University, Changsha, Hunan, China

**Keywords:** senescence, mTOR, GTPase, liver cancer, chemoresistance

## Abstract

Radiotherapy and chemotherapy can arrest cancer cells in a senescence-like state, which can lead to therapy resistance and cancer relapse. mTOR is hyperactivated in senescent cells but the mechanisms remain unclear. In this study, we examine the roles of several mTOR-regulated GTPases in senescence-like liver cancer cells and the mechanisms in drug resistance. We show that although RagC, Rheb, Rab1A, Rab5 and Arf1 GTPases were required for optimal mTOR activation in proliferating HepG2 cells, only RagC and Rheb are required in the senescence-like counterparts. Consistently, the drug resistance of the senescence-like HepG2 can be reduced by knocking down RagC and Rheb but not the other GTPases. Autophagic and lysosomal activity were increased in senescence-like cells; pharmacological inhibition of autophagy-lysosome decreased mTOR activity and preferentially sensitized senescence-like HepG2 cells to chemotherapy drugs including trametinib, cisplatin, and doxorubicin. In liver cancer patients, expression of RagC and Rheb but not other GTPases examined was associated with unfavorable prognosis. Our study therefore has defined a key role of Rag-Rheb GTPase in mediating mTOR activation and drug resistance in senescence-like HepG2 cells, which could have important implications in developing second-line treatments for liver cancer patients.

## Introduction

Senescence is traditionally defined as an irreversible cell cycle arrest in G1 phase (G1 exit) triggered by eroded telomeres in aged primary cells (Campisi and d’Adda di Fagagna, 2007; Childs et al., 2017). Senescent cells show elevated β-galactosidase (β-GAL) activity, which is frequently used as a marker to identify cellular senescence (Dimri et al., 1995). Many other cellular perturbations can also cause senescence or senescence-like phenotype, for example, abnormal chromatin organization, proteostasis, mitochondrial respiration (Pazolli et al., 2012; Moreno et al., 2019; Joy et al., 2021). In cancer cells, chemotherapy and radiotherapy can induce oxidative and genotoxic stress, triggering a senescence-like state (Collado and Serrano, 2006; Di Micco et al., 2006). Cancer cells in the senescence-like state are known to cause treatment resistance in many types of cancer (Gordon and Nelson, 2012; Thompson et al., 2021). These senescence-like cancer cells, through unknown mechanisms, support tumor cell proliferation, leading to cancer recurrence (Gordon and Nelson, 2012; Thompson et al., 2021). Targeting senescence-like cancer cells therefore promises better treatments, but the mechanisms regulating the senescence-like state remain unclear.

mTOR has been shown to participate in senescence establishment (Xu et al., 2014). mTOR is a Serine/Threonine protein kinase regulating multiple aspects of cell growth and maintenance, including protein synthesis, autophagy, mitochondrial function (Wei et al., 2015; Mossmann et al., 2018). In response to nutrient signals, including growth hormone, glucose, or amino acid (AA), mTOR is rapidly activated and phosphorylates effector proteins for signal transduction (Hay and Sonenberg, 2004). S6 Kinase is one such effector among others, whose phosphorylation at Threonine-389 (S6K-T389) is frequently used to assay mTOR activity (Holz and Blenis, 2005). Persistent mTOR activity is associated with drug resistance in cancer treatments, including Mitogen-activated protein kinase kinase (MEK) inhibitors (Murugan, 2019; Wang et al., 2021). In senescent cells, mTOR was persistently elevated and insensitive to serum and AA starvation (Zhang et al., 2000; Carroll et al., 2017). How mTOR is persistently activated and whether it is related to senescence-induced drug resistance has not been sufficiently investigated.

A serial of small GTPases including Rheb, Rag, Rab1, Rab5, Arf1, and GTPase have been shown to regulate mTOR activity in response to nutrients cues. Rag is protein complex consisting of two sets of heterodimers: Rag A/B and Rag C/D GTPase, knocking down of Rag C subunit is sufficient to block the function of the whole complex (Sancak et al., 2010). RagC and Rheb functions in the same pathway to modulate AA sensing. In response to AA stimulation, Rag complex recruits mTOR to the lysosomal membrane, facilitating its activation by the Rheb GTPase (Groenewoud and Zwartkruis, 2013). Interestingly however, several other studies showed that the Rag GTPases and homologs such as yeast Gtr1/2 and mouse RagC are not essential for AA sensing, as cells lacking the Rag complex remain sensitive to AA stimulation and activate mTOR, albeit less efficiently (Efeyan et al., 2014; Stracka et al., 2014; Jewell et al., 2015; Thomas et al., 2016). Rab1A GTPase is a well-known regulator of protein trafficking between ER and Golgi. Rab1A was found to bind and activate mTOR in response to AA stimulation (Thomas et al., 2016). In a genetic screen, the ER-Golgi associated GTPase, Arf1 and the endosome-associated GTPase, Rab5 were found to activate mTOR in response to AA stimulation (Li et al., 2010), suggesting diverse mechanisms in mTOR regulation under different physiological conditions. Whether and how these GTPases regulate mTOR in senescent cells or senescence-like cancer cells remains poorly characterized.

## Materials and methods

### Cell culture and drug treatments

HepG2 cells were obtained from The Cell Bank of Type Culture Collection of Chinese Academy of Sciences. All experiments were performed in accordance with institutional guidelines and regulations. Cells were maintained on petri dish in Dulbecco’s Modified Eagle Medium (DMEM) supplemented with 10% (vol/vol) fetal bovine serum (FBS), 100 units/ml penicillin, and 100 μg/ml streptomycin in a 37°C incubator with 5% CO_2_ and moisture. Senescence-like cells were obtained by exposing HepG2 cells to 20 Gy X-ray on petri dish. Fresh medium was added immediately after irradiation then every 3 or 4 days. Senescence was induced for 2 weeks. Starvation was conducted by replacing culture medium with AA and serum-free DMEM for 1 h. For partial AA starvation, cells were changed to AA-free DMEM then supplemented AA Solution (ThermoFisher) to 5% of suggested concentration for 18 h. MEK inhibitor trametinib was obtained from Sigma and dissolved in DMSO as 10 mM stock, then diluted in cell culture the final concentration of 10 nM for 3 h. Lysosome inhibitors Bafilomycin A1 (BAF) and hydroxychloroquine (HCQ) and were added to cells at the final concentration of 100 nM and 100 uM, respectively, for 3 h.

### siRNA knockdown

siRNAs were custom designed and purchased from ThermoFisher Scientific. Cells were transfected with siRNA using Lipofectamine 3000 (ThermoFisher Scientific) according to the manufacturer’s instructions. HepG2 cells were irradiated and maintained in culture medium for 2 weeks to induce senescence before siRNA transfection. Briefly, siRNA and lipofectamine 3000 were separately diluted in Opti-MEM medium for 5 min and mixed. After 20 min incubation at RT, siRNA and lipofectamine mix were added to proliferating and senescent HepG2 cells at 90% confluency. The medium was changed after 24 h. Except for Rab5, the siRNA for RagC, Rheb, and Arf1 are pools of two oligoes. The sequences were shown in Supplementary Table S1. The knockdown effects of individual and combined siRNAs were shown in Supplementary Figure S1.

### Western blotting

Cells on petri dish were washed with PBS then lysed with 1X SDS-PAGE sample loading buffer diluted from 4X loading buffer (250 mM Tris-HCl pH 6.8; 8% SDS; 0.2% Bromophenol Blue; 20% β-mercaptoethanol; 40% glycerol). Whole cell lysates were heated at 95°C for 5 min and separated by SDS-PAGE and transferred to polyvinylidene fluoride (PVDF) membrane. Western blotting was conducted by blocking the membranes in 5% non-fat milk then probing with primary antibodies in 5% non-fat milk for 1 h. Membranes were washed extensively with PBST (phosphate buffered saline supplemented with 0.5% Tween 20), then incubated with HRP-conjugated secondary antibodies for 30 min. After extensive wash with PBST, bound HRP-conjugated secondary antibody was detected by enhanced chemiluminescence (ECL). Antibodies against human mTOR (ab2732), mTOR-S2481 (ab137133), S6K1 (ab9366), S6K-T389 (ab2571), RAGC (ab226199), RAB1A (ab241132), RAB5 (ab18211), and Tubulin (ab15568) were purchased from Abcam. Antibody against LC3 (848801) was from BioLegend.

### Flow cytometry

Flow cytometry was used to quantify senescence, apoptotic cell death, and lysosomal activity. For senescence, 1E5 cells were washed with ice-cold Flow Buffer (PBS+0.5% BSA) then fixed in 4% formaldehyde for 15 min at room temperature. After permeabilization with 0.5% Triton X-100 in PBS, cells were incubated with β-galactosidase substrate CellEvent™ Senescence Green and Click-iT Plus EdU (measuring cell proliferation) for 1 h in the absence of CO_2_. Cells were washed in Flow buffer three times and analyzed on cytoFLEX S (BECKMAN) using a 488-nm laser and 530-nm/30 filter. Apoptotic cell death was measured by staining cells with Annexin V (indictor for early apoptosis) and propidium iodide (PI, indicator for cell death), according to the manufacturer’s instructions (Sigma). Briefly, cells were collected into 1.5 ml Eppendorf tube by trypsin, then blocked in Flow Buffer (PBS supplemented with 1% BSA) for 1 h. Cells were then incubated with FITC-conjugated Annexin V for 15 min. Cells were washed with Flow Buffer extensively to remove non-specific binding. PI was added at the final concentration of 1 ug/ml. Lysosomal Intracellular Activity Assay Kit (Abcam) was used to measure lysosomal activity according to the product manual. Briefly, cells were incubated with Self-Quenched Substrate for 1 h in medium supplemented with 0.5% FBS. Cells were washed in 1X PBS containing and analyzed on a flow cytometer (488 nm excitation laser).

### Cell viability assay

Cell viability was done by CellTiter-glo reagent (Promega) according to manufacturer’s manual. Briefly, HepG2 cells on 96-well plate treated with indicated drugs were removed of culture medium and lysed for 10 min at room temperature with rapid shaking, then luminescent ATP detecting reagent was added and incubated in the dark for 20 min, then read on plate reader. After subtracting the background signal, signal for drug treated samples were normalized to non-treated sample as 100%. Experiments were performed 3–3 times, four replicates for each time.

### TCGA data analysis

LIHC patient data were originally from TCGA database and were analyzed on the informatics platform GEPIA (www.gepia.cancer-pku.cn). Anonymous data were acquired and analyzed in accordance with institutional guidelines and regulations. For differential gene expression, |Log2FC| ≥ 0.5 and *p* < 0.01 were used as criteria and compared to Match TCGA normal and GTEx data. Data were presented as Boxplots for indicated genes. To compare the prognosis of LIHC patients with high (top 25%) and low (low 25%) expression of indicated genes, overall survivals were plotted using Kaplan Meier curve. *p* values were derived from the Log-Rank test.

### Statistics

All experiments were performed multiple times and tested by unpaired, two-tailed Student’s *t*-test or two-way ANOVA as indicated. For flow cytometry, cells were gated with the same standard for all samples in each experiment. Data from at least three experiments were analyzed with Prism 7 software. For Western blotting, signals were quantified by ImageJ software and normalized to tubulin control. Data were further normalized to experimental controls (100%) for each experiment and fold changes of three experiments were analyzed with Prism 7. Significance was defined by *p* < 0.01.

## Results

### Rag and Rheb GTPase but not Rab1A, Rab5, or Arf1 were required for persistent mTOR activity in senescence-like hepG2 cells

**FIGURE 1:**
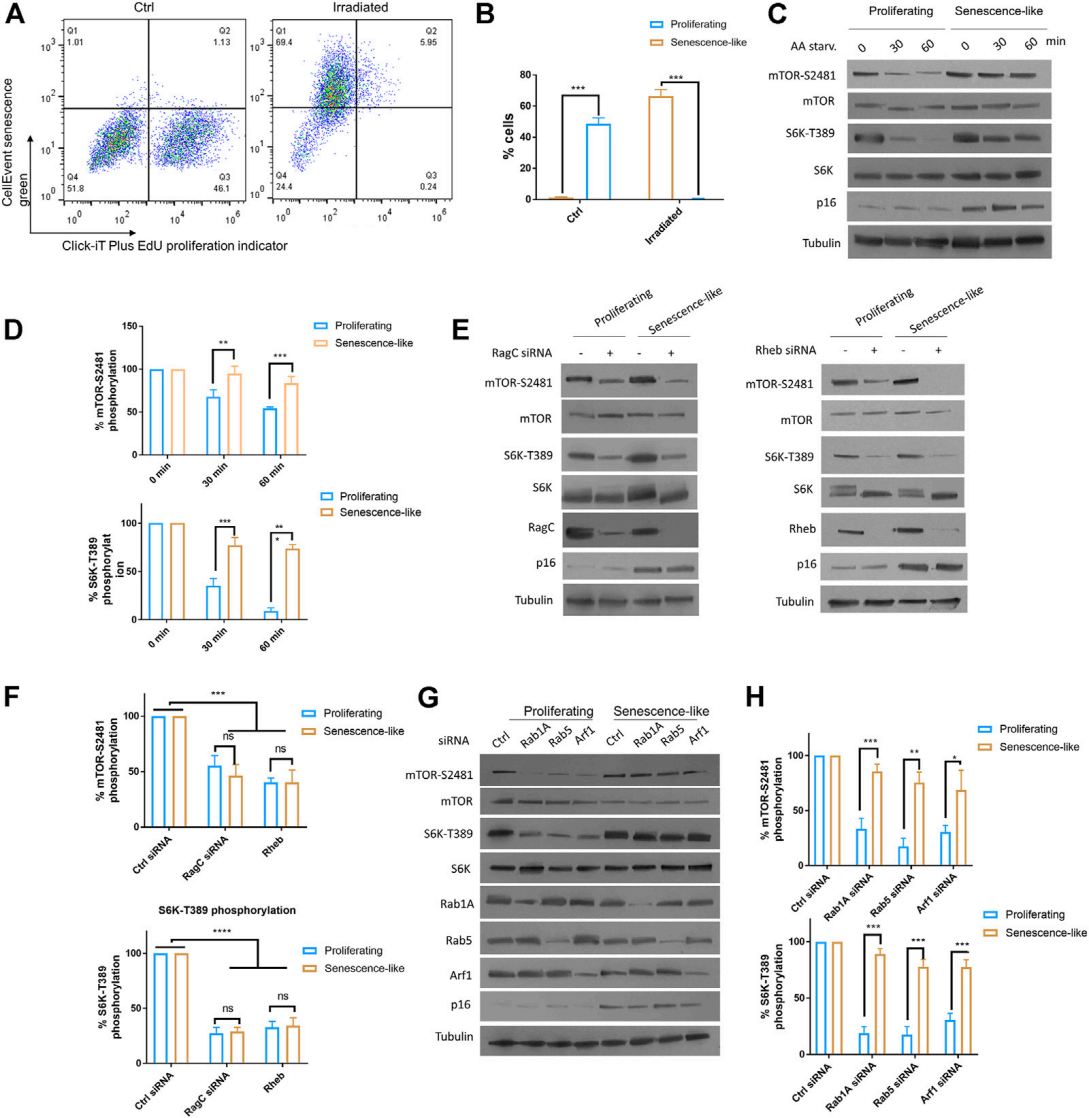
Rag and Rheb but not Rab1A, Rab5, Arf1 were required for persistent mTOR activity in senescence-like HepG2 cells. **(A)** HepG2 cells were irradiated with 20 Gy X-ray and maintained on fresh medium over 2 weeks. Non-irradiated cells and irradiated cells were examined for senescence marker (CellEvent senescence green) and proliferating marker (Click-iT Plus EdU) by flow cytometry. All samples were gated through the same standard. Representative data are shown. **(B)** Quantification of senescence-like cell populations from 3 separate experiments. *p* < 0.001(***). **(C)** mTOR activity was insensitive to AA starvation in senescence-like HepG2 cells. Proliferating and senescence-like HepG2 cells were cultured in medium without AA and serum for 30 min or 1 h. Total proteins were separated by reduced SDS-PAGE. mTOR-S2481 and S6K-T389 phosphorylation were examined by Western blotting. Representative data are shown. **(D)** Quantification of mTOR activity by mTOR-S2481 and S6K-T389. Blots were quantified by ImageJ and normalized to control (100%) for each experiment. N = 3 experiments were tested by Student’s *t*-test. *p* < 0.001(***). **(E)** Persistent mTOR activity was dependent on Rag and Rheb GTPases. RagC and Rheb were knocked down by siRNA for 48 h mTOR-S2481 and S6K-T389 were examined by Western blotting. Representative data are shown. **(F)** Quantification of multiple experimental results in **(E)**. Blots were quantified by ImageJ and normalized to control (100%) for each experiment. N = 3 experiments were tested by Student’s *t*-test. *p* < 0.0001(****), ns, not significant. **(G)** mTOR activity was insensitive to knockdowns of Rab1A, Rab5 and Arf1 expression in senescence-like HepG2 cells. Gene expressions were knocked down by transfecting cells with specific siRNAs for 48 h. Indicated proteins were examined by Western blotting. Representative data are shown. **(H)** Quantification of mTOR-S2481 and S6K-T389 phosphorylation in **(G)**. Blots were quantified by ImageJ and normalized to control (100%) for each experiment. N = 3 experiments were tested by Student’s *t*-test. *p* < 0.001(***).

We aimed to characterize the roles of small GTPase activators of mTOR including RagC, Rab1A, Rab5, and Arf1 in senescence-like hepatoma cell line HepG2. We first applied x-ray radiation to HepG2 cells to obtain senescence-like cells. After 2 weeks post-radiation, cells are arrested and showed significant β-galactosidase activity, as determined by flow cytometry using CellEvent Senescence Green probe and cell proliferating maker Click-iT EdU (Figures 1A,B). Second, we examined mTOR activity in response to AA starvation by Western blotting of two well established markers: mTOR and S6 kinase phosphorylation (mTOR-S2481 and S6K-T389, respectively). The results showed that in senescence-like HepG2 cells, mTOR activity was much higher than proliferating cells after 30 and 60 min of AA starvation (Figures 1C,D). Third, we knocked down RagC, Rheb, Rab1A, Rab5, and Arf1 through specific siRNAs in senescence-like and proliferating HepG2 cells. After senescence induction, the HepG2 cells can be readily transfected with siRNA, as shown by Western blotting (Figures 1E–H). All these GTPase knockdowns reduced mTOR activity in proliferating cells. Interestingly however, in the senescence-like HepG2 cells, RagC and Rheb but not Rab1A, Rab5, and Arf1 siRNA significantly decreased S6K-T389 levels (Figures 1E–H), suggesting that RagC and Rheb GTPase were specifically required for mTOR activity in the senescence-like state. RagC is one of the four Rag GTPases, knocking down of RagC did not affect the expression of RagA, RagB, or RagD (Supplementary Figure S2). Therefore, RagC’s effect is not likely mediated by other Rag GTPases.

### Knocking down RagC and Rheb but not Rab1A, Rab5, or Arf1 increased drug sensitivity in senescence-like HepG2 cells

**FIGURE 2:**
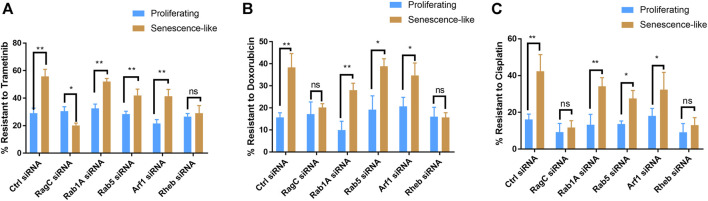
Knocking down Rag and Rheb but not Rab1A, Rab5, Arf1 increased drug sensitivity in senescence-like HepG2 cells. **(A)** RagC and Rheb were required for Trametinib resistance in senescence-like HepG2 cells. Indicated GTPase expression were knocked down by siRNA in proliferating and senescence-like HepG2 cells and drug was added. Viability was determined by CellTiter-glo after 3 days Trametinib (10 nM) treatment. Data were normalized to untreated controls for each experiment. **(B)** RagC and Rheb were required for Doxorubicin resistance in senescence-like HepG2 cells. HepG2 cells of senescence-like and proliferating counterparts were treated with 100 nM Doxorubicin for 3 days and survival were quantified by CellTier-glo. **(C)** RagC and Rheb were required for cisplatin resistance in senescence-like HepG2 cells. HepG2 cells of senescence-like and proliferating counterparts were treated with 1 µM Cisplatin for 3 days and survival were quantified by CellTier-glo. N = 3 experiments were plotted and analyzed by Student’s *t* test *p* < 0.01(**), *p* < 0.05(*).

We asked if RagC could be also specifically required for drug resistance in senescence-like HepG2 cells. To this aim, we treated senescence-like HepG2 cells and proliferating controls with 10 nM trametinib, a well-known MEK inhibitor for 3 h. We then examined apoptosis and cell death through staining cells with Annexin V and propidium iodide (PI), respectively, followed by flow cytometry analysis (Supplementary Figure S3A). Consistently with previous studies, senescence-like cells were much resistant to the MEK inhibitor than proliferating cells. With the siRNA treatments, only RagC knockdown significantly increased the sensitivity to the MEK inhibitor (Supplementary Figure S3B). We quantified the PI-stained cell death in short-term (3 h) Trametinib treatment. Interestingly, statistical analysis of N = 3 experiments showed no resistance of senescence-like HepG2 cells to chemotherapy drug Trametinib (Supplementary Figure S3C). We therefore further studied the effect of gene knockdowns on long term drug resistance. Long-term survival was examined by CellTiter-glo after treating cells with 10 nM trametinib, 100 nM Doxorubicin, or 1 µM Cisplatin for 3 days. Consistently, senescence-like HepG2 cells were significantly more resistant to these chemotherapy drugs than proliferating counterparts. Interestingly, knocking down of RagC or Rheb but not the other GTPases canceled the resistant phenotype of senescence-like HepG2 cells (Figures 2A–C). The results suggest that Rag and Rheb GTPase but not Rab1A, Rab5, or Arf1 GTPases were required for drug resistance phenotype in senescence-like HepG2 cells.

### Hydroxychloroquine and bafilomycin-A1 reduced mTOR activity and sensitized senescence-like HepG2 cells to a MEK inhibitor

**FIGURE 3:**
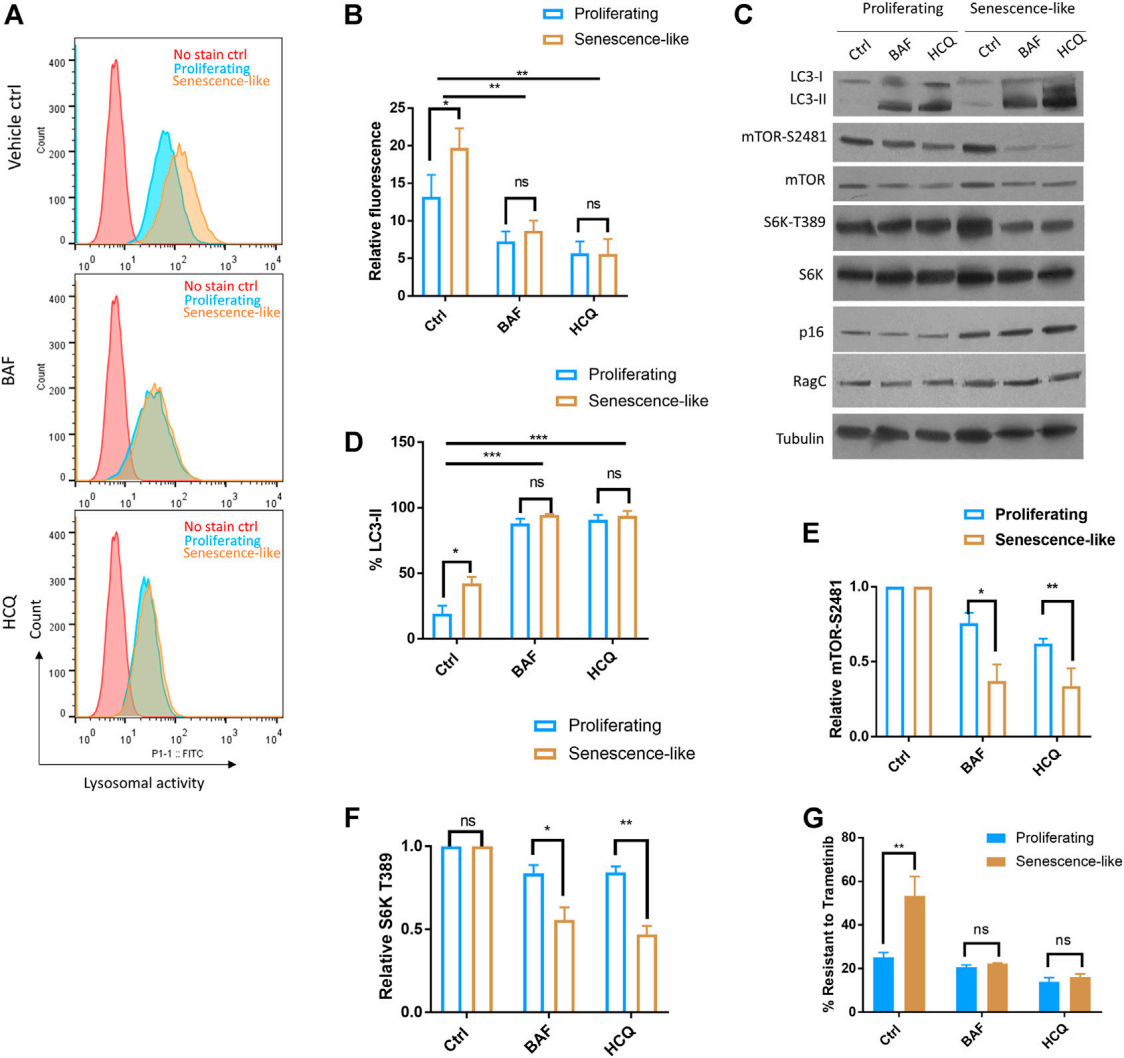
Hydroxychloroquine and Bafilomycin-A1 reduced mTOR activity and sensitized senescence-like HepG2 cells to a MEK inhibitor. **(A)** Flow cytometry confirmed the inhibition of lysosomal activity by bafilomycin A1 (BAF) and hydroxychloroquine (HCQ). Cells treated with 100 nM BAF and 100 uM HCQ for 3 h were stained with a self-quenched fluorescent substrate from Lysosomal Intracellular Activity Assay Kit (Abcam). Senescence-like HepG2 cells showed elevated lysosomal activity. No stained controls were used as references. Shown are representative data. **(B)** Quantification of three experimental data shown in **(A)**. Student’s *t* test *p* < 0.05(*), 0.01(**), not significant (ns). **(C)** HCQ and BAF reduced mTOR activity in senescence-like but not proliferating HepG2 cells. Cells were treated with 100 nM BAF and 100 uM HCQ for 3 h and indicated proteins were examined by Western blotting. Accumulation of LC3-II indicated the lack of lysosomal degradation. Shown are representative data. **(D)** Quantification of LC3-II accumulation in N = 3 experiments. Accumulation of LC3-II indicated the lack of lysosomal degradation. Student’s *t* test *p* < 0.05(*), 0.001(***), not significant (ns). **(E)** Quantification of mTOR activity by mTOR-S2481 phosphorylation. Blots were quantified by ImageJ and normalized to control (100%). N = 3 experiments were tested by Student’s *t* test *p* < 0.05(*), 0.01(**), not significant (ns). **(F)** Quantification of mTOR activity by S6K-T389 phosphorylation. Blots were quantified by ImageJ and normalized to control (100%). N = 3 experiments were tested by Student’s *t* test *p* < 0.05(*), 0.01(**), not significant (ns). **(G)** BAF and HCQ treatment sensitize senescence-like HepG2 cells to MEK inhibitor Trametinib. Cells were treated with 10 nM Trametinib and100 nM BAF or 100 uM HCQ for 3 days and survival were examined by CellTiter-glo. Data were normalized to untreated controls for each experiment. N = 3 experiments were plotted and analyzed by Student’s *t* test *p* < 0.01(**), ns, not significant.

The Rag complex is localized to the lysosome and plays an essential role in amino acid sensing and mTOR activation. To test if lysosome is involved, we first examined if autophagic and lysosomal activity was elevated in senescence-like HepG2 cells. By incubating cells with a lysosome-specific self-quenching substrate (which will emit fluorescence when degraded by lysosomal proteases), our flow cytometry experiments showed that lysosomal activity was robustly increased upon senescence induction (Figures 3A,B). Second, we inhibited autophagic and lysosomal activity by hydroxychloroquine (HCQ) and Bafilomycin-A1 (BAF). Both drugs successfully reduced lysosomal activity as revealed by flow cytometry and accumulation of autophagic marker LC3 proteins (Figures 3A–D). For both inhibitors, 1-h treatment already reduced mTOR activity in the senescence-like HepG2 cells but had only slight effects on the proliferating counterparts (Figures 3E,F). Consistently, both autophagy-lysosome inhibitors sensitized the senescence-like HepG2 cells to the MEK inhibitor Trametinib, but only had a slight effect on the proliferating HepG2 counterpart (Figure 3G). Together, our results suggested that senescence-like cancer cells had increased dependency on autophagy-lysosome for nutrient supply and cellular maintenance. Despite the lack of specificity, HCQ and BAF have been commonly used to study the effect of autophagy-lysosome inhibition on many biological processes (Mauthe et al., 2018). However, since HCQ and BAF could also affect post-Golgi and endosome vesicle trafficking (Mauthe et al., 2018; Chen and Geiger, 2020) and the latter have been known to regulate mTOR activity (Flinn et al., 2010; Makhoul and Gleeson, 2021), our experiments did not rule out the involvement of Golgi or endosome in modulating mTOR activity and drug resistance in senescence-like HepG2 cells.

### Elevated lysosomal activity increased mTOR activity in a RagC-dependent manner

**FIGURE 4:**
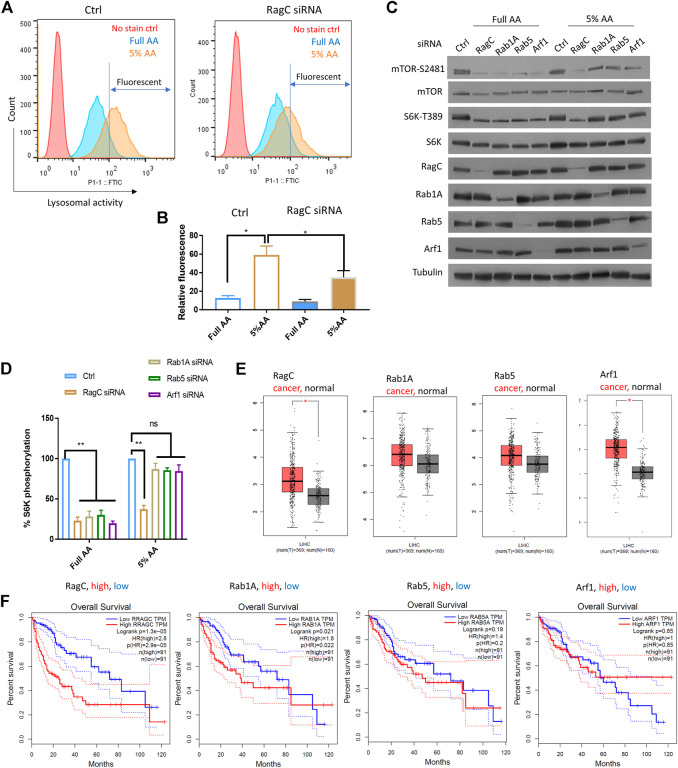
RagC activated mTOR in response to elevated lysosomal activity and predicted poor survival in LIHC patients. **(A)** AA starvation increased lysosomal activity in a RagC-dependent manner. HepG2 cells were knocked down of RagC expression by siRNA then starved in culture medium with 5% of normal AA for 18 h, stained with lysosomal activity probe and analyzed by flow cytometry. **(B)** Quantification of N = 2 experiments in **(A)**. Student’s *t* test *p* < 0.05(*). **(C)** mTOR activity was sensitive to RagC knockdown but not Rab1A, Rab5, or Arf1 knockdown in HepG2 cells with elevated lysosomal activity (induced by 5% AA starvation). Indicated proteins were examined by Western blotting. Representative data were shown. **(D)** Quantification of Western blot data in **(C)**. Blots were quantified by ImageJ and normalized to control (100%). N = 3 experiments were tested by Student’s *t* test *p* < 0.01(**), not significant (ns). **(E)** Expression of indicated genes in N = 369 LIHC cancer patients and N = 160 matched normal controls. *p* < 0.01(*). LIHC patient data from TGCA were analyzed with GEPIA bioinformatics tools. **(F)** Expression of RagC but not other GTPases is a robust prognostic predictor for LIHC patients. Overall Survival (OS) data of LIHC patients with top 25% high (N = 91) and 25% low (N = 91) expression of indicated genes were plotted in Kaplan Meier curves and tested by Log-rank test. *p* < 0.01 was considered significant. HR, hazard ratio. TPM, transcript per million.

To further confirm the role of Rag complex in senescence-like HepG2 cells, we chemically activate lysosomal activity and test the dependency of mTOR on RagC. As RagC and Rheb are known to function in the same pathway for AA sensing (Groenewoud and Zwartkruis, 2013), we focused only on RagC. To test this, we applied a partial AA starvation strategy, allowing for examination of S6K-T389 that was difficult to detect in full starvation. By decreasing the AAs to 5% of the normal concentration in the medium for 18 h, we found a significant increase in lysosomal activity in HepG2 cells compared to those cultured in full AA (Figures 4A,B). We knocked down RagC, Rab1A, Rab5, and Arf1 by siRNA and examined mTOR-S2481 and S6K-T389 levels by Western blotting. All siRNA knockdowns fully inhibited mTOR-S2481 and S6K-T389 phosphorylation in normally cultured HepG2 cells (Figures 4C,D). Under partial starvation however, only RagC siRNA robustly decreased mTOR-S2481 and S6K-T389 levels; other siRNAs showed much reduced effects. Therefore, the results confirmed that cells with elevated autophagic and lysosomal activity had increased dependency on Rag GTPase for mTOR activation, lending further supports to the specific functions of Rag GTPase in promoting mTOR activity and drug resistance in senescence-like HepG2 cancer cells.

### RagC was stronger than several GTPases as a prognostic predictor of unfavorable outcome in LIHC patients

We investigated the clinical data regarding the association of the GTPases with liver cancer and the overall survival of Liver Hepatocellular Carcinoma (LIHC) patients. RNAseq and survival data from the NIH The Cancer Genome Atlas (TCGA) database were analyzed by using GEPIA, a web-based bioinformatics tool (Tang et al., 2017). By examining the differential expression of RagC, Rheb, RalA, Rab1A, Rab5, and Arf1 in cancer tissues (n = 369) and non-cancer controls (*n* = 160), we showed that RagC and Arf1 but not Rheb, RalA, Rab1A or Rab5 were significantly elevated in cancer tissues (Figure 4E, Supplementary Figure S4). The LIHC patients had gone through different treatments including chemotherapy and radiotherapy, which induced senescence-like cancer cells. If RagC but not other mTOR regulating GTPases was specifically important for maintaining the survival and drug resistance of the senescence-like HepG2 cells, then higher levels of RagC but not other GTPases should predict poor survival. Indeed, by plotting the Kaplan Meier curve for LIHC patients with the 25% highest and 25% lowest RagC expression, we showed that RagC -high patients had significantly worse overall survival (Figure 4F). The hazard ratio was 2.8, indicating that the RagC-high patients were 2.8 times more likely to die than RagC-low patients. By similar standards, higher expression of Rab1A, Rab5, and Arf1 did not show significant (*p* < 0.01) different survival than lower expressing controls. Although the expression of Rheb is not significantly higher in LIHC group, its overexpression is significantly associated with poor cancer prognosis (Supplementary Figure S4). As RagC functions to recruit mTOR to Rheb in the lysosomal membrane for activation, this result is consistent with the important role of RagC in utilizing lysosomal nutrients in senescent state. Interesting, RalA expression is also correlated to poor survival (Supplementary Figure S4). Whether and how RalA is involved in mTOR regulation of senescent-like cancer cell remains to be studied.

## Discussion

Senescent cells are arrested in the cell cycle and are resistant to many cancer treatments, including chemotherapy and radiotherapy (Campisi and d’Adda di Fagagna, 2007; Childs et al., 2017; Gordon and Nelson, 2012; Thompson et al., 2021). The underlying mechanisms remain not well understood. mTOR is a key regulator of tumor growth and its persistent activity has been reported to cause chemo-resistance and radio-resistance (Murugan, 2019). By comparing several small GTPase regulators of mTOR in proliferating and senescence cells, we show that RagC and Rheb, but not Rab1A, Rab5, or Arf1 is required for the persistent mTOR activity in senescence-like HepG2 cells. Consistently, RagC complex but not the other GTPases increases the resistance to cancer drugs trametinib, a potent MEK inhibitor currently under clinical trials for several cancer types. We found that RagC complex promoted mTOR activity in response to elevated lysosomal activity. Chemicals that inhibit autophagy-lysosome pathway restored the sensitivity of HepG2 cells to the MEK inhibitor. Our study has important implications in developing better strategies for liver cancer treatments.

Recently, mTOR has been proposed to mainly regulate AA sensing through RagC/D GTPase (Sancak et al., 2010). Interestingly, mTOR is localized to multiple subcellular organelles, such as Golgi, ER, mitochondria, endosome, and lysosome (Betz and Hall, 2013; Carroll, 2020). mTOR has also been shown to bind directly to the nuclear chromatin (Wei et al., 2009; Kantidakis et al., 2010). The broad localization patterns suggest that mTOR could sense nutrients including AA through multiple sites. Consistently, mTOR was regulated by multiple small GTPases including Rheb, RagC, Rab1A, Rab5, RalA, and Arf1 (Li et al., 2010; Sancak et al., 2010; Thomas et al., 2016). Our study in senescence-like cancer cells showed that in AA-rich medium all GTPases were required for optimal mTOR activity. However, in senescence-like cells where autophagic and lysosomal activity were elevated, RagC and Rheb GTPase but not other GTPases examined affect AA-stimulated mTOR activity. RagC is known to recruit mTOR to the lysosomal membrane and facilitate the interaction with Rheb (Groenewoud and Zwartkruis, 2013), it is therefore consistent that Rheb and RagC behave similarly in senescence-like HepG2 cells. Why senescence-like HepG2 cells specifically prefer RagC complex rather than other GTPase? One explanation is that HepG2 cells can sense AA through multiple subcellular locations when cultured in rich medium. When entering the senescence-like state, HepG2 cells predominantly relies on AA supplies from autophagy-lysosomal degradation of internal cellular components. As RagC complex is mostly localized to the lysosome membrane, its function become essential for senescence-like cancer cells. Further study on Rag complex could make it a ideal druggable target for treating senescence-induced chemoresistance in liver cancer patients.

Senescence-like cancer cells develop multiple ways to escape treatments (Wang et al., 2022). One mechanism is by entering a dormant state where growth and proliferation are mostly halted, causing resistance to drugs that target proliferating cells (Recasens and Munoz, 2019). In our case, the radiation-induced senescence-like state shows elevated mTOR activity, suggesting that cells were not dormant. Another known mechanism of drug resistance is the modulation of the tumor microenvironment by releasing SASP. SASP has been known to release cell factors and inflammatory regulators to promote drug resistance (Chambers et al., 2021). The mechanisms remain poorly understood but could involve modulation of tumor microenvironment and promoting cancer metastasis (Chambers et al., 2021). In our current study, RagC knockdown increased apoptotic cell death (Supplementary Figure S3) and at the same time increased drug sensitivity (Figure 2). Therefore, we favor a potential mechanism whereby RagC/D-mTOR modulates lysosomal activity and cell death to regulate drug resistance. Given the pivotal role of mTOR in senescence, future studies may investigate if there is a direct connection between mTOR activity and SASP.

Our study has several limitations. First, we have compared only a few GTPases, while mTOR is regulated by many other GTPases directly or indirectly. Without further investigations, it remains unclear if RagC is the only mTOR regulator of AA sensing in senescence-like cells. Second, we have been focused only in HepG2 cells, where senescence was robustly induced by irradiation. Whether the differential requirement of RagC complex in senescence-like cancer cells in other type of cancers remains unclear. Third, although widely used to inhibit autophagy-lysosome, HCQ and BAF also affect post-Golgi and endosome vesicle trafficking (Mauthe et al., 2018; Chen and Geiger, 2020). Therefore, the resulted mTOR inhibition and chemo-sensitizing effect upon HCQ and BAF treatment only suggest a correlation to RagC complex, and the potential role of lysosome in senescence-like HepG2 cells awaits further investigations.

## Data Availability

The original contributions presented in the study are included in the article/Supplementary Material, further inquiries can be directed to the corresponding author.
