# Supplementary material for: RagC GTPase regulates mTOR to promote chemoresistance in senescence-like HepG2 cells
Source: Front Physiol. 2022 Oct 4;13:949737. doi: 10.3389/fphys.2022.949737 (PMC9577253; doi:10.3389/fphys.2022.949737)
Supplement: Supplementary file 1 [file DataSheet1.PDF]

Supplementary materials

Wei Jiang [1,2], Zhenglin Ou [1,2], Qin Zhu [1,2], Hongyan Zai [1,2,*]

Table S1 siRNA sequence used in this study

Protein	ID	Sequence	Ref.
**RagC**	#1	UAGACUACAACUUCCACUG	
	#2	UCUACAUUGCAACAGACAG	
**Rheb**	#1	UCAGUGUAGUUUGUUGUUUAA	1
	#2	UCUUGUGUAUUCUGUUACA	
**Rab5**	#1	GCCAAUUUCAUGAAUUUCAUU	2
	#2	CAGCCAUAGUUGUAUAUGAUU	2
**Arf1**	#1	GAAAUGCGCAUCCUCAUGGUGG	3
	#2	CCACGAUCCUCUACAAGCU	
**Rab1A**	#2	AAUAACUGGAGGUGAUUGUUC	4

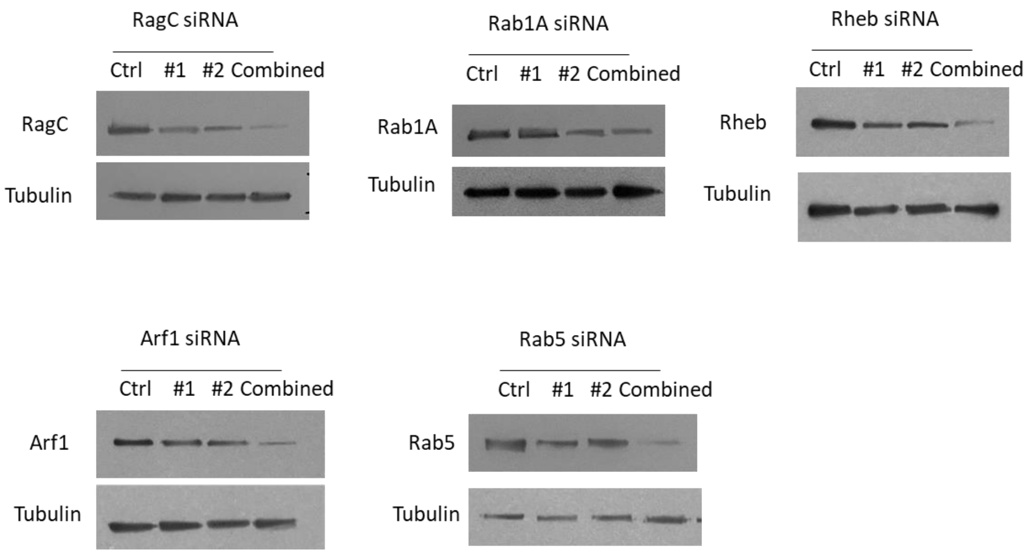

Figure S1 Effect of individual and combined siRNAs on gene expression. HepG2 cells were irradiated with 20 Gy X-ray and maintained on fresh medium over 2 weeks. 48 hours after indicated siRNA (sequence in table S1) were transfected, cells were homogenized, and crude protein were separated by SDS-PAGE. knockdown efficiencies were checked by western blotting.

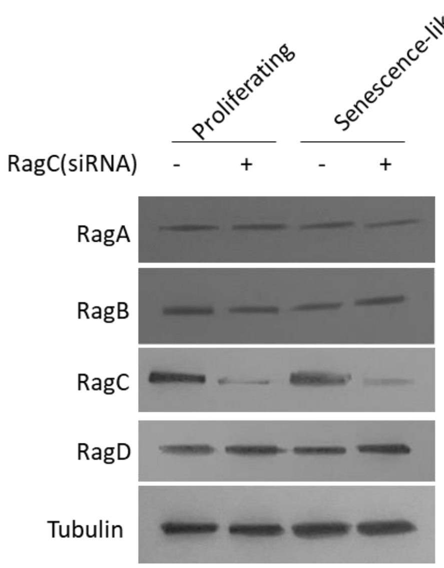

Fig S2 RagC siRNA did not affect RagA, RagB, and RagD expression.
HepG2 cells were irradiated with 20 Gy X-ray and maintained on fresh medium over 2 weeks. 48 hours after RagC siRNA were transfected, cells were homogenized, and crude lysate were separated by SDS-PAGE. RagA, RagB, RagC, and RagD expression were examined by western blotting.

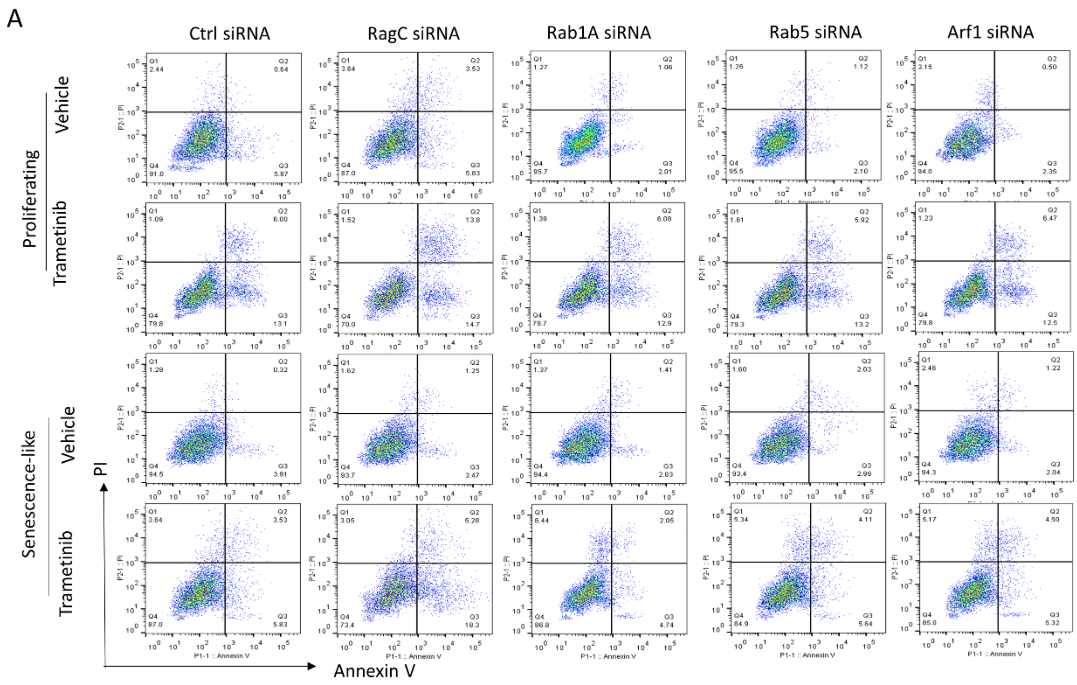

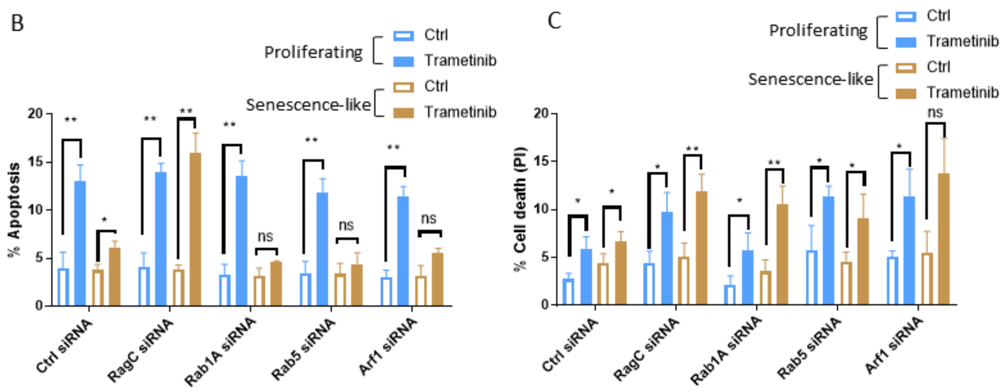

Figure S3 GTPase knockdown effect on short-term Trametinib treatment of proliferating and senescence-like HepG2 cells. (A) Indicated GTPase expression were knocked down by siRNA in proliferating and senescence-like HepG2 cells, then cells were treated with 10 nM Trametinib for 3 hrs. Apoptosis and cell death were examined by staining cells with Annexin-V and propidium iodide (PI) followed by flow cytometry. Representative data are shown. **(B)** % apoptosis was quantified for 2 experiments and statistically analyzed by Student t test. P<0.05(*), 0.01(**), not significant (ns). **(C)** Cell death were examined by staining cells with Annexin-V and propidium iodide (PI) followed by flow cytometry. Statistical analysis of N= 3 experiment shows no resistance of senescence-like HepG2 cells to chemotherapy drug Trametinib, therefore no way to analysis of the GTPases knockdown on drug resistance.

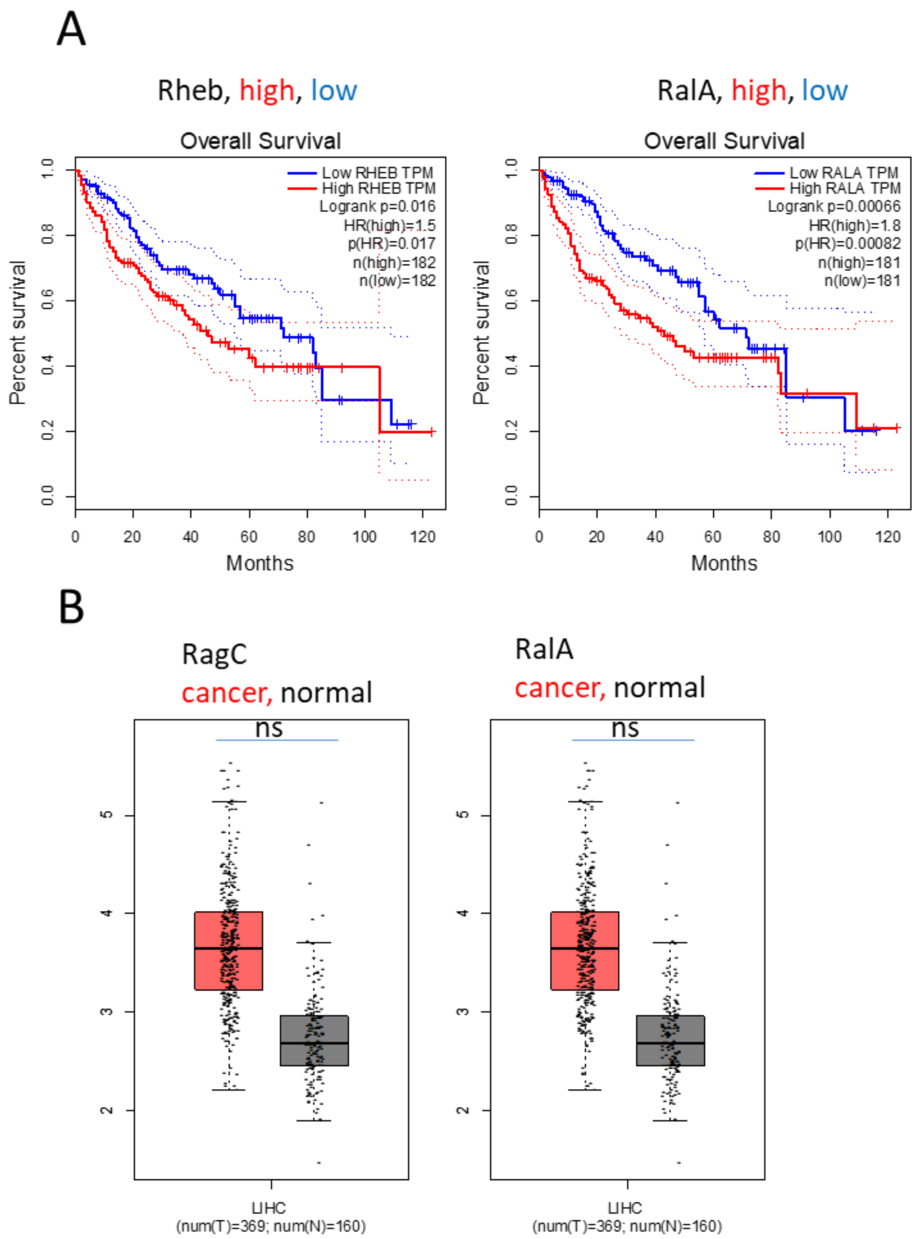

Figure S4 Rheb and RalA expression is associated with poor prognosis of LIHC patients. **(A)** Overall Survival (OS) data of LIHC patients with top 50% high (N = 182) and 50% low (N=182) expression of indicated genes were plotted in Kaplan Meier curves and tested by Log-rank test. P <0.01 was considered significant. HR, hazard ratio. TPM, transcript per million. **(B)** Expression of indicated genes in N= 369 HepG2 cancer patients and N =160 matched normal controls. P<0.01(*). LIHC patient data from TGCA were analyzed with GEPIA bioinformatics tools.

References:

1 Long, X., Lin, Y., Ortiz-Vega, S., Busch, S. & Avruch, J. The Rheb switch 2 segment is critical for signaling to target of rapamycin complex 1. *J Biol Chem* **282**, 18542-18551, doi:10.1074/jbc.M610736200 (2007).

2 Chan, L. *et al.* Role of Rab5 in the formation of macrophage-derived foam cell. *Lipids Health Dis* **16**, 170, doi:10.1186/s12944-017-0559-6 (2017).

3 Boulay, P. L., Cotton, M., Melancon, P. & Claing, A. ADP-ribosylation factor 1 controls the activation of the phosphatidylinositol 3-kinase pathway to regulate epidermal growth factor-dependent growth and migration of breast cancer cells. *J Biol Chem* **283**, 36425-36434, doi:10.1074/jbc.M803603200 (2008).

4 Wang, X. *et al.* Expression of Rab1A is upregulated in human lung cancer and associated with tumor size and T stage. *Aging (Albany NY)* **8**, 2790-2798, doi:10.18632/aging.101087 (2016).
